# Real-world Experience of Bezlotoxumab for Prevention of *Clostridioides difficile* Infection: A Retrospective Multicenter Cohort Study

**DOI:** 10.1093/ofid/ofaa097

**Published:** 2020-03-19

**Authors:** Richard L Hengel, Timothy E Ritter, Ramesh V Nathan, Lucinda J Van Anglen, Claudia P Schroeder, Ryan J Dillon, Stephen W Marcella, Kevin W Garey

**Affiliations:** 1 Atlanta ID Group, Atlanta, Georgia; 2 GI Alliance, Southlake, Texas, USA; 3 Mazur, Statner, Dutta, Nathan, PC, Thousand Oaks, California, USA; 4 Healix Infusion Therapy, LLC., Sugar Land, Texas, USA; 5 Merck & Co., Inc., Kenilworth, New Jersey, USA; 6 University of Houston College of Pharmacy, Houston, Texas

**Keywords:** bezlotoxumab, *Clostridioides difficile* infection, outpatient, recurrence

## Abstract

**Background:**

Bezlotoxumab is approved for prevention of recurrence of *Clostridioides difficile* infection (CDI) in adults receiving standard of care (SoC) therapy based on findings from MODIFY clinical trials. However, utilization practices and validation of trial results in the real world are limited.

**Methods:**

Records of patients receiving bezlotoxumab between April 2017 and December 2018 across 34 infusion centers in the United States were retrospectively reviewed. Recurrent CDI (rCDI), defined as diarrhea lasting ≥2 days resulting in treatment, was assessed 90 days postbezlotoxumab.

**Results:**

The study cohort included 200 patients (median age, 70 years; 66% female; median Charlson comorbidity index, 5), of whom 86% (n = 173) had prior CDI episodes and 79% (n = 158) had ≥2 risk factors for rCDI. SoC antibiotics included vancomycin (n = 137, 68%), fidaxomicin (n = 60, 30%), and metronidazole (n = 3, 2%). Median time from *C. difficile* stool test to bezlotoxumab and initiation of SoC to bezlotoxumab were 15 days and 11 days, respectively. Within 90 days, 31 of 195 patients (15.9%) experienced rCDI, which corresponds to a success rate of 84.1%. Patients with ≥2 CDI recurrences prebezlotoxumab had a higher risk of subsequent rCDI compared with those with 1 recurrence or primary CDI (hazard ratio, 2.77; 95% confidence interval, 1.14–6.76; *P = *.025).

**Conclusions:**

This real-world multicenter study demonstrated successful prevention of rCDI with bezlotoxumab comparable to clinical trial results regardless of type of SoC and timing of infusion. Multiple prior CDI recurrences were associated with a higher risk of subsequent rCDI, supporting the use of bezlotoxumab earlier in the disease course.

*Clostridioides difficile* causes ~500_ _000 infections in the United States and is responsible for 44_ _000 deaths and $5.4 billion in health care costs annually [1]. Up to 25% of patients with initial *C. difficile* infection (CDI) who successfully respond to standard of care (SoC) antibiotic therapy with oral metronidazole or vancomycin will experience recurrences; fewer recurrences are observed for patients given fidaxomicin [2–4]. Patients with recurrent CDI (rCDI) are at an even higher risk for subsequent recurrences and more severe disease associated with increasing morbidity and risk for mortality [5–7]. The management of CDI patients with multiple recurrences remains a challenge due to limited therapeutic options [8, 9].

Bezlotoxumab (Zinplava, Merck & Co., Inc., Kenilworth, NJ, USA) is a human monoclonal antibody approved for the prevention of rCDI in patients ≥18 years of age receiving concomitant SoC antibiotic therapy for CDI and who are at high risk for recurrence [10]. In 2 large randomized, double-blind, placebo-controlled phase 3 trials, MODIFY I and II, patients who received bezlotoxumab in combination with SoC therapy had a significantly lower rate of rCDI at 12-week follow-up compared with those who received SoC therapy alone (MODIFY I: 17% vs 28%; *P < *.001; MODIFY II: 15% vs 26%; *P < *.001) [11]. Analysis of pooled trial data indicated that bezlotoxumab was particularly effective in patients with ≥1 risk factor for rCDI, with the greatest benefit in those with ≥3 risk factors including age ≥65 years, history of CDI in the previous 6 months, compromised immunity, severe CDI, and having a strain associated with poor outcomes of CDI [12].

 Data demonstrating prevention of rCDI with bezlotoxumab in a real-world setting are limited. A recent observational study of 46 patients from 5 hospitals in Finland reported that bezlotoxumab prevented rCDI in 73% of patients [13]. Our study evaluates the CDI recurrence rate in patients receiving bezlotoxumab in combination with SoC antibiotic therapy across US outpatient infusion centers. Patient characteristics, utilization practices, and known risk factors for rCDI were assessed.

## METHODS

### Study Design, Study Population, and Data Source

This retrospective, multicenter cohort study included patients receiving bezlotoxumab between April 2017 and December 2018 in geographically diverse US outpatient infusion centers. All infusion centers were part of infectious disease or gastroenterologist physician offices and were managed by an infusion center management company using centralized electronic databases. The study was approved by an independent institutional review board (IntegReview IRB, Austin, TX, USA).

Adults with primary CDI or rCDI receiving SoC antibiotic therapy were included. Data collection from medical records included patient demographics, prior hospitalization within 4 weeks of the current CDI episode, history of CDI prebezlotoxumab, and fecal microbiota transplant (FMT). Comorbidity burden was assessed using the Charlson comorbidity index. History of congestive heart failure (CHF) was recorded. Risk factors associated with rCDI included age ≥65 years, compromised immunity, current CDI episode with severe presentation, and ≥1 CDI episode within the past 6 months [11, 12]. Based upon Infectious Disease Society of America/Society for Healthcare Epidemiology of America and hospital-specific guidelines, a CDI episode was considered severe if any of the following were present at the time of diagnosis: albumin ≤3 g/dL, serum creatinine ≥1.5 times baseline, hypotension or shock, intensive care unit stay related to CDI, ileus, serum lactate >5 mmol/L, toxic megacolon or colectomy related to CDI, and white blood cell count ≥15_ _000 cells/μL [9, 14]. Compromised immunity was defined as use of immunosuppressive medication and/or presence of underlying disease (immune deficiency, history of solid organ or hematopoietic stem cell transplant, absolute neutrophil cell count <500 cells/μL) [9]. Additional characteristics collected included use of gastric acid suppressants (histamine-2 receptor blocker, proton pump inhibitor) [5, 13, 15], use of non-CDI antibiotics within 4 weeks of current CDI episode [5, 17, 18], chronic renal disease [19], and inflammatory bowel disease [20].

Characterization of utilization practices included SoC antibiotic at the time of bezlotoxumab use and median time intervals (days) from (i) positive *C. difficile* stool test to bezlotoxumab and (ii) initiation of SoC to bezlotoxumab. Bezlotoxumab (10 mg/kg) was administered as a single intravenous infusion over 60 minutes. Diagnostic methods used for *C. difficile* confirmation were captured. Additional information regarding outpatient use of bezlotoxumab was collected (Supplementary Tables 1 and 2).

Recurrent CDI was defined as recurrence of diarrhea lasting 2 or more days resulting in medical intervention (SoC antibiotic and/or FMT) with or without positive stool test for toxigenic *C. difficile*. According to the study protocol, rCDI was assessed ≥90 days postbezlotoxumab by physician follow-up visit and/or scripted telephone interview with the patient or caregiver. Severity, subsequent hospitalization due to rCDI, and treatment postbezlotoxumab were documented. Categorical baseline characteristics and outcomes between this real-world study and MODIFY I and II (MODIFY) trial data were compared [11, 12, 22]. No safety end points were specified in the protocol; however, serious adverse events and deaths were reported to Merck & Co., Inc. (Kenilworth, NJ, USA).

### Statistical Analysis

Descriptive statistics inclusive of frequencies and percentages for categorical data and mean (SD), or median (range) for continuous data were used to describe the patient population and utilization characteristics. The Kaplan-Meier method was used to describe the cumulative probability (%) of rCDI stratified by CDI history prebezlotoxumab and analyzed using the log-rank chi-square test. Risk factors for rCDI were assessed using univariate (Student *t* test and Pearson chi-square) and multivariate Cox proportional hazards regression models [21]. Hazard ratio (HR) and 95% confidence interval (CI) were tabulated, with a *P* value <.05 considered statistically significant. All statistical analyses were performed using SAS, version 9.4 (SAS Institute, Cary, NC, USA).

## RESULTS

### Real-world Study Population

A total of 200 patients from 34 US physician infusion centers received bezlotoxumab in combination with SoC for prevention of rCDI. Demographics and clinical characteristics are shown in Table 1. The median age (range) was 70 (21–98) years, and 131 patients (65.5%) were female. Before receiving bezlotoxumab, 73 patients (36.5%) were hospitalized within 4 weeks of their current CDI episode for a mean duration of 5 ± 4 days, the majority (n = 67) due to CDI. The median Charlson comorbidity index score (range) was 5 (3–9). At baseline, 27 patients (13.5%) had primary CDI, 50 (25.0%) had 1 recurrence ever, 62 (31.0%) had 2 recurrences ever, and 61 (30.5%) had ≥3 CDI recurrences ever. The distribution of rCDI risk factors included age ≥65 years (n = 134, 67.0%), compromised immunity (n = 84, 42.0%), current CDI with severe presentation (n = 56, 28.0%), and ≥1 CDI episode in the past 6 months (n = 154, 77.0%). Overall, 158 patients (79.0%) had ≥2 and 65 patients (32.5%) had ≥3 of these 4 risk factors. Other characteristics included 86 patients (43.0%) on gastric acid suppressants, 58 (29.0%) on non-CDI antibiotics within 4 weeks before current CDI, 35 (17.5%) with chronic renal disease, 23 (11.5%) with previously failed FMT, 18 (9.0%) with inflammatory bowel disease, and 10 (5.0%) with a history of CHF.

**Table 1.** Baseline Demographics and Clinical Characteristics of Patients Receiving Bezlotoxumab in Outpatient Infusion Centers

Characteristic	Results (n = 200)
Age, y	
Mean (SD)	67 (15)
Median (range)	70 (21–98)
Female	131 (65.5)
Hospitalization within 4 wk of current CDI	73 (36.5)
Mean length of stay (SD), d	5 (4)
Charlson comorbidity index, median (range)	5 (3–9)
CDI history	
Primary CDI	27 (13.5)
No. of CDI recurrences ever	
1	50 (25.0)
2	62 (31.0)
≥3	61 (30.5)
rCDI risk factor	
Age ≥65 y	134 (67.0)
Compromised immunity^a^	84 (42.0)
Current CDI with severe presentation^b^	56 (28.0)
≥1 CDI episode in the past 6 mo	154 (77.0)
No. of rCDI risk factors per patient^c^	
0	3 (1.5)
≥2	158 (79.0)
≥3	65 (32.5)
Other characteristics	
Gastric acid suppressant use^d^	86 (43.0)
Non-CDI antibiotic within 4 wk before current CDI	58 (29.0)
Chronic renal disease^e^	35 (17.5)
Prior failed FMT	23 (11.5)
Inflammatory bowel disease	18 (9.0)
History of congestive heart failure	10 (5.0)

Data are presented as No. (%) unless otherwise indicated.

Abbreviations: CDI, *Clostridioides difficile* infection; FMT, fecal microbiota transplant; rCDI, recurrent *Clostridioides difficile* infection.

^a^Due to immunosuppressive medication or underlying disease (immune deficiency, solid organ or hematopoietic stem cell transplant, absolute neutrophil cell count <500 cells/μL).

^b^Defined by any of the following: albumin ≤3.0 g/dL, serum creatinine ≥1.5 times above baseline, hypotension or shock, intensive care unit stay related to CDI, ileus, serum lactate >5 mmol/L, toxic megacolon or colectomy related to CDI, white blood cell count ≥15_ _000 cells/μL.

^c^Age ≥65 years, immunocompromised state, current CDI with severe presentation, and CDI episodes experienced in the past 6 months.

^d^Proton pump inhibitor and histamine-2 receptor antagonist.

^e^Defined as serum creatinine ≥1.5 mg/dL.

Characteristics related to bezlotoxumab utilization in the outpatient setting are shown in Table 2. Diagnostic tests for *C. difficile* confirmation included polymerase chain reaction (PCR) method (n = 153, 76.5%) and toxin enzyme immunoassay (EIA; n = 47, 23.5%). Oral SoC antibiotics included vancomycin fixed dose (n = 76, 38.0%), vancomycin tapered regimen (n = 61, 30.5%), fidaxomicin (n = 60, 30.0%), and metronidazole (n = 3, 1.5%), all prescribed in combination with bezlotoxumab. The median lengths of SoC therapy of vancomycin fixed dose, vancomycin tapered regimen, fidaxomicin, and metronidazole were 14 days, 42 days, 10 days, and 14 days, respectively. The median time intervals from positive *C. difficile* stool test to bezlotoxumab and initiation of SoC to bezlotoxumab (range) were 15 (2–97) days and 11 (2–144) days, respectively.

**Table 2.** Oral Standard-of-Care Antibiotic Therapy and Bezlotoxumab Utilization

Characteristic	Results (n = 200)
Diagnostic test method for *C. difficile* confirmation	
PCR	153 (76.5)
Toxin EIA	47 (23.5)
SoC antibiotic at time of bezlotoxumab, No. of patients (%)	
Vancomycin fixed dose^a^	76 (38.0)
Vancomycin tapered regimen	61 (30.5)
Fidaxomicin	60 (30.0)
Metronidazole	3 (1.5)
Length of SoC therapy, median (range), d	
Vancomycin fixed dose	14 (9–170)
Vancomycin tapered regimen	42 (15–168)
Fidaxomicin	10 (9–32)
Metronidazole	14 (14–30)
Bezlotoxumab utilization parameter	
Days from *C. difficile* stool test to bezlotoxumab, median (range)	15 (2–97)
Days from initiation of SoC to bezlotoxumab, median (range)^b^	11 (2–144)

Data are presented as No. (%) unless otherwise indicated.

Abbreviations: *C. difficile*, *Clostridioides difficile*; EIA, enzyme immunoassay; PCR, polymerase chain reaction; SoC, standard of care.

^a^Including 125 mg (n = 40), 200 mg (n = 1), 250 mg (n = 28), and 500 mg (n = 7).

^b^Thirty-nine of 76 patients (51.3%) received vancomycin fixed dose >10 days, 5 of 61 patients (6.6%) received vancomycin taper >8 weeks, and 18 of 60 patients (30.0%) received fidaxomicin >10 days [9].

During the study period, requests for reimbursement for 144 additional patients were submitted to third-party payors for approval of bezlotoxumab that ultimately did not receive the infusion (Supplementary Table 1). Primary reasons were payor denial (n = 65, 45.1%), competing problem (n = 24, 16.7%), and patient decision (n = 19, 13.2%) (Supplementary Table 2).

### CDI Recurrence

**Figure 1.**
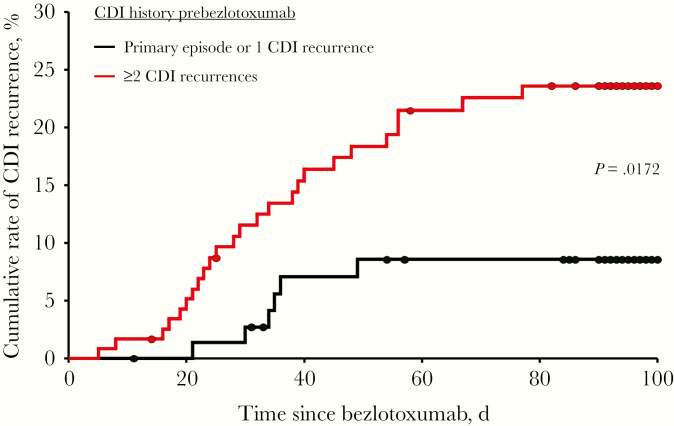
Kaplan-Meier plot representing time to recurrence of *Clostridioides difficile* infection (CDI) in patients receiving bezlotoxumab stratified by CDI history prebezlotoxumab. Day 0 is defined as the day of bezlotoxumab infusion. The difference between patients with a primary episode or 1 CDI recurrence and patients with ≥2 CDI recurrences was statistically significant using the log-rank chi-square test (*P = *.0172).

Recurrence was assessed in 195 of 200 patients, with 31 patients (15.9%) experiencing rCDI within 90 days. All patients had recurrent diarrhea leading to medical intervention, of whom 23 had a positive *C. difficile* stool test (22 PCR, 1 EIA), and 8 patients had no additional testing performed. Postbezlotoxumab, 17 (55%) patients with rCDI were treated with SoC antibiotics, 12 (39%) with SoC and FMT, and 2 (6%) with FMT only. Hospitalization due to rCDI occurred in 11 of 31 patients, 3 with severe disease. The median time to recurrence (range) was 31 (5–76) days. Fifteen patients (48%) experienced rCDI within 30 days, 14 (45%) between 30 and 60 days, and 2 (7%) between 60 and 90 days postbezlotoxumab, respectively. Summary statistics for baseline variables comparing recurrent patients (n = 31) and nonrecurrent patients (n = 164) are shown in Table 3. The proportion of female patients was higher in the recurrent group vs the nonrecurrent group; however, this was not statistically significant (74.2% vs 62.8%; *P = *.225). A Charlson comorbidity index ≥3 and the type of oral SoC antibiotic at the time of bezlotoxumab did not affect recurrence rates. Based on the SoC regimen used, recurrence rates for vancomycin fixed dose, vancomycin taper, and fidaxomicin were 13.7%, 18.3%, and 15.2%, respectively. In the metronidazole group, 1 of 3 patients (33%) had a CDI recurrence. Significantly more patients in the rCDI group had a history of ≥2 CDI recurrences ever compared with patients in the nonrecurrent group (80.6% vs 57.9%; *P = *.017). The distribution of risk factors was not significantly different between recurrent and nonrecurrent patients. Likewise, the numbers of CDI risk factors per patient were comparable. The recurrence rate for patients with previously failed FMT was comparable to that of those without FMT (11.3% vs 15.0%). Other characteristics and utilized diagnostic test methods demonstrated no significant differences between the recurrent and nonrecurrent groups. Time to recurrence stratified by CDI history prebezlotoxumab is shown in Figure 1. Using the Cox proportional hazards model, patients with ≥2 CDI recurrences were independently associated with a higher risk for subsequent rCDI compared with those with only 1 recurrence or primary CDI (hazard ratio [HR], 2.77; 95% confidence interval [CI], 1.14–6.76; *P = *.025).

**Table 3.** Summary Statistics for Categorical Baseline Variables Comparing Recurrent CDI Patients and Nonrecurrent Patients After a Single Dose of Bezlotoxumab

Categorical Variable	All (n = 195)	Recurrent CDI		Proportion With CDI Recurrence, no./No. (%)	*P* Value
		Yes (n = 31)	No (n = 164)		
Female	126 (64.6)	23 (74.2)	103 (62.8)	23/126 (18.2)	.225
Charlson comorbidity index ≥3	147 (75.4)	22 (70.9)	125 (76.2)	22/147 (14.9)	.531
SoC antibiotic at time of bezlotoxumab					
Vancomycin, fixed dose	73 (37.4)	10 (32.3)	63 (38.4)	10/73 (13.7)	.514
Vancomycin, tapered regimen	60 (30.8)	11 (35.5)	49 (29.9)	11/60 (18.3)	.536
Fidaxomicin	59 (30.2)	9 (29.0)	50 (30.5)	9/59 (15.2)	.868
Metronidazole	3 (1.6)	1 (3.2)	2 (1.2)	1/3 (33.3)	.405
CDI history					
Primary CDI	26 (13.3)	1 (3.2)	25 (15.2)	1/26 (3.8)	.072
No. of CDI recurrences ever					
1	49 (25.1)	5 (16.1)	44 (26.8)	5/49 (10.2)	.209
≥2	120 (61.5)	25 (80.6)	95 (57.9)	25/120 (20.8)	.017
rCDI risk factor					
Age ≥65 y	132 (67.7)	19 (61.3)	112 (68.3)	19/132 (14.4)	.448
Compromised immunity^a^	81 (41.5)	13 (41.9)	69 (42.1)	13/81 (16.0)	.984
Current CDI with severe presentation^b^	53 (27.2)	9 (29.0)	46 (28.0)	9/53 (16.9)	.909
≥1 CDI episode in the past 6 mo	151 (77.4)	27 (87.1)	123 (75.0)	27/151 (17.9)	.143
No. of rCDI risk factors per patient^c^					
0	3 (1.5)	0 (0)	3 (1.8)	0/3 (0)	.453
1	37 (18.9)	7 (22.6)	30 (18.3)	7/37 (18.9)	.576
≥1	192 (98.5)	31 (100)	161 (98.2)	31/192 (16.1)	.453
2	93 (47.7)	14 (45.2)	79 (48.2)	14/93 (15.0)	.759
≥3	62 (31.8)	10 (32.2)	52 (31.7)	10/62 (16.1)	.956
Other characteristics					
Gastric acid suppressant use^d^	81 (41.5)	16 (51.6)	65 (39.6)	16/81 (19.8)	.215
Non-CDI antibiotic use within prior 4 wk	54 (27.7)	5 (16.1)	49 (29.8)	5/54 (9.2)	.118
Chronic renal disease^e^	33 (16.9)	5 (16.1)	28 (17.1)	5/33 (15.2)	.892
Inflammatory bowel disease	17 (8.7)	4 (12.9)	13 (7.9)	4/17 (23.5)	.366
Prior failed FMT	22 (11.3)	5 (16.1)	17 (10.4)	5/22 (22.7)	.359
No prior FMT	173 (88.7)	26 (83.9)	147 (89.6)	26/173 (15.0)	.359
Diagnostic test method					
PCR	149 (76.4)	26 (83.8)	123 (75.0)	26/149 (17.4)	.291
Toxin EIA	46 (23.6)	5 (16.1)	41 (25.0)	5/46 (10.8)	.286

Data are presented as No. (%). *P* value compares recurrent CDI group vs nonrecurrent CDI group using Pearson’s chi-square test.

Abbreviations: CDI, *Clostridioides difficile* infection; EIA, enzyme immunoassay; FMT, fecal microbiota transplant; PCR, polymerase chain reaction; SoC, standard of care.

^a^Due to immunosuppressive medication or underlying disease (immune deficiency, solid organ or hematopoietic stem cell transplant, absolute neutrophil count <500 cells/μL).

^b^Defined by any of the following: albumin ≤3.0 g/dL, serum creatinine ≥1.5 times above baseline, hypotension or shock, intensive care unit stay related to CDI, ileus, toxic megacolon or colectomy related to CDI, serum lactate >5 mmol/L, white blood cell count ≥15_ _000 cells/μL.

^c^Age ≥65 years, immunocompromised state, current CDI with severe presentation, and CDI episodes experienced in the past 6 months.

^d^Proton pump inhibitor and histamine-2 receptor antagonist.

^e^Defined as serum creatinine ≥1.5 mg/dL.

No infusion-related reactions were reported for patients receiving bezlotoxumab. Five patients had no outcomes reported due to loss of follow-up (n = 3) and death (n = 2). One death was reported for an 80-year-old female with a history of worsening interstitial lung disease related to rheumatoid arthritis and CHF, who presented to the hospital with respiratory failure and health care–associated pneumonia 1 week postbezlotoxumab. She received bezlotoxumab following her 5th CDI episode and died 75 days after the infusion. The other death occurred in a 31-year-old female with multiple medical problems including systemic lupus erythematosus, end-stage renal disease, CHF, and myocarditis status post–mitral valve replacement who received bezlotoxumab after her 6th CDI episode. She developed generalized weakness with significant edema in her lower extremities 6 days after bezlotoxumab infusion, which was attributed to nonfunctional peritoneal dialysis. This patient died 40 days postbezlotoxumab.

### Comparison Between Real-world and MODIFY

Table 4 compares categorical baseline characteristics and CDI recurrence rates between this real-world study cohort (n = 195) and patients who received bezlotoxumab in the pooled MODIFY trials (n = 781) [11, 12, 22]. The median ages were comparable between both study populations (71 years vs 66 years; *P = *.169). Significantly more female patients were recruited in the real-world study compared with MODIFY (64.6% vs 56.6%; *P = *.042). In addition, our study had significantly more patients with a Charlson comorbidity index ≥3 (75.4% vs 40.8%; *P < *.0001) and significantly more patients with ≥2 CDI recurrences (61.5% vs 12.8%; *P < *.0001). Conversely, fewer patients received bezlotoxumab following treatment of a primary CDI episode compared with patients in MODIFY trials (13.3% vs 54.3%; *P < *.0001). Significantly fewer patients in the real-world study received vancomycin fixed dose compared with MODIFY (37.4% vs 47.6%; *P < *.0106). A tapered course of vancomycin, excluded in MODIFY trials, was given to 30.8% of patients. The use of fidaxomicin was higher (real world: 30.2%; vs MODIFY: 3.8%; *P < *.0001) and the use of metronidazole was lower (real world: 1.6%; vs MODIFY: 48.5%; *P < *.0001) than in clinical trials.

**Table 4.** Comparison Between Real-world Study and MODIFY I and II Phase 3 Clinical Trials^^a^^

Parameter	Real-world Cohort (n = 195)	MODIFY I/II^a^Bezlotoxumab Cohort (n = 781)	*P* Value
Age, median (range), y	71 (21–98)	66 (18–100)	.169
Female	126 (64.6)	442 (56.6)	.042
Charlson comorbidity index ≥3	147 (75.4)	319 (40.8)	<.0001
Hospitalization at time of referral	70 (35.9)	530 (67.9)	<.0001
CDI history			
Primary CDI	26 (13.3)	424 (54.3)	<.0001
No. of CDI recurrences ever			
1	49 (25.1)	150 (19.2)	.067
≥2	120 (61.5)	100 (12.8)	<.0001
SoC antibiotic at time of bezlotoxumab			
Vancomycin, fixed dose	73 (37.4)	372 (47.6)	<.0106
Vancomycin, tapered regimen	60 (30.8)	0	-
Fidaxomicin	59 (30.2)	30 (3.8)	<.0001
Metronidazole	3 (1.6)	379 (48.5)	<.0001
rCDI risk factor			
Age ≥65 y	132 (67.7)	390 (49.9)	<.0001
Compromised immunity^b^	81 (41.5)	178 (22.8)	<.0001
Current CDI with severe presentation^c^	53 (27.2)	122 (15.6)	.0002
≥1 CDI episodes in the past 6 mo	151 (77.4)	216 (27.6)	<.0001
No. of rCDI risk factors per patient^d^			
0	3 (1.6)	189 (24.2)	<.0001
1	37 (18.9)	283 (36.2)	<.0001
≥2	155 (79.5)	307 (39.3)	<.0001
Other characteristics			
Chronic renal disease^e^	33 (16.9)	123 (15.7)	.682
Chronic liver disease^f^	8 (4.1)	49 (6.3)	.242
Prior failed FMT	22 (11.3)	0	-
Diagnostic method PCR or toxigenic culture	149 (76.4)	399 (51.1)	<.0001
CDI recurrence at 12-wk follow-up			
All patients	31 (15.9)	129 (16.5)	.839
Patients with ≥1 rCDI risk factor^d^	31/192 (16.1)	100/471 (21.2)	.135

Data are presented as No. (%) unless otherwise indicated. *P* value compares real-world vs MODIFY I/II study cohorts using a 2-sided *t* test and Pearson’s chi-square test.

Abbreviations: CDI, *Clostridioides difficile* infection; FMT, fecal microbiota transplant; PCR, polymerase chain reaction; SoC, standard of care.

^a^Pooled modified intention-to-treat population according to Wilcox et al. [11] and Gerding et al. [12].

^b^Due to immunosuppressive medication or underlying disease (immune deficiency, solid organ or hematopoietic stem cell transplant, absolute neutrophil count <500 cells/μL).

^c^Defined by any of the following: albumin ≤3.0 g/dL, serum creatinine ≥1.5 times above baseline, hypotension or shock, intensive care unit stay related to CDI, ileus, toxic megacolon or colectomy related to CDI, serum lactate >5 mmol/L, white blood cell count ≥15_ _000 cells/μL.

^d^Age ≥65 years, compromised immunity, current CDI with severe presentation, and CDI episodes experienced in past 6 months. Note, hypervirulent *Clostridioides difficile* strains were not available to assess as a CDI risk factor in this study; however, they were included in the MODIFY I/II trials.

^e^Defined as serum creatinine ≥1.5 mg/dL.

^f^Defined as mild, moderate, or severe liver disease as reported on the Charlson comorbidity index.

Risk factors for rCDI analyzed in both studies, real world vs MODIFY, indicated significant differences for age ≥65 years (67.7% vs 49.9%; *P < *.0001), compromised immunity (41.5% vs 22.8%; *P < *.0001), current CDI with severe presentation (27.2% vs 15.6%; *P = *.0002), and ≥1 CDI episode in the past 6 months (77.4% vs 27.6%; *P < *.0001). Significantly more patients in this real-world cohort had ≥2 rCDI risk factors compared with MODIFY participants (79.5% vs 39.3%; *P < *.0001). Similar frequencies were obtained for patients with chronic renal disease (real world: 16.9%; vs MODIFY: 15.7%; *P = *.682) and chronic liver disease (real world: 4.1%; vs MODIFY: 6.3%; *P = *.242). The PCR method was more frequently used in this real-world study than in MODIFY trials (76.4% vs 51.1%; *P < *.0001). CDI recurrence rates were comparable between real-world vs MODIFY for all patients (15.9% vs 16.5%; *P = *.839) and for patients with ≥1 rCDI risk factor (16.1% vs 21.2%; *P = *.135).

## DISCUSSION

Bezlotoxumab has been shown to effectively reduce the risk of rCDI in the pivotal MODIFY trials [11]. However, real-world evidence in clinical practice settings is limited [13]. This study was conducted with a comprehensive network of infusion clinics throughout the US, providing the largest real-world cohort receiving bezlotoxumab in the outpatient setting to date. Our findings indicate that a single infusion of bezlotoxumab resulted in a CDI recurrence rate of 15.9%, which is comparable to 16.5% reported for the overall population enrolled in the MODIFY trials [11].

This real-world cohort had a larger proportion of patients at risk for rCDI compared with the MODIFY population and a higher proportion with multiple rCDI risk factors. The majority of patients (86%) had at least 1 CDI recurrence prebezlotoxumab. Approximately 80% of patients had ≥2 risk factors for rCDI, most frequently ≥1 CDI episode in the past 6 months, and age ≥65 years. In addition to a high at-risk patient population, multiple comorbidities were present at baseline, as shown by the median Charlson comorbidity index of 5. Risk analysis revealed that having ≥2 CDI recurrences prebezlotoxumab was a significant predictor for subsequent rCDI, suggesting that bezlotoxumab use during earlier episodes may improve outcomes. Patients with ≥2 CDI recurrences prebezlotoxumab had a higher rate of subsequent rCDI (20.8%) as compared with those with 1 recurrence ever (10.2%) or a primary CDI (3.8%).

In contrast to MODIFY trials, rCDI was clinically defined as recurrence of diarrhea for ≥2 days leading to medical intervention regardless of the availability of a positive *C. difficile* stool test. This could have resulted in a greater proportion of diarrhea recurrences classified as rCDI, as confirmatory diagnostic tests were not consistently performed in this study. In addition, documentation of hypervirulent types of *C. difficile* strains was not available for all patients, and therefore could not be assessed as an rCDI risk factor. Vancomycin and fidaxomicin were the primary SoC antibiotics compared with almost half of patients receiving metronidazole in the MODIFY trials. The difference in antibiotic therapy may have been influenced by updated guidelines for the management of CDI after the MODIFY trials, by a larger number of patients with multiple rCDIs who previously failed courses of metronidazole, and/or by a greater number of patients with severe CDI episodes, for which metronidazole is not recommended [9]. The use of a fixed or tapered dose vancomycin or fidaxomicin as the SoC antibiotic did not affect the recurrence rate, whereas the use of oral metronidazole as SoC was too infrequent to draw any conclusions. Furthermore, the recurrence rate of patients with previously failed FMT was comparable to that of those without FMT. This is the first reported use of bezlotoxumab in patients who received a tapered vancomycin SoC regimen or had previously failed FMT, as both cohorts were excluded from the MODIFY trials.

The overall CDI recurrence rate of 15.9%, which corresponds to a success rate of 84.1%, is better than expected considering that the real-world population in comparison with MODIFY was comprised largely of patients who had failed multiple prior courses of varied CDI therapies. Therefore, it appears more appropriate to compare the outcomes of patients with at least 1 rCDI risk factor, as these individuals more closely resemble the population receiving bezlotoxumab in MODIFY trials. The recurrence rates of patients with ≥1 rCDI risk factor were 16.1% for the real-world cohort and 21.2% for the bezlotoxumab group in the MODIFY trials [12]. For comparison, the recurrence rate for patients with ≥1 risk factor who received SoC without bezlotoxumab (ie, placebo group) in MODIFY was 37.2% [12].

Compared with clinical trial data, longer delays from *C. difficile* stool test to administration of bezlotoxumab and initiation of SoC to bezlotoxumab were observed [11, 22]. On average, bezlotoxumab was administered 3 days after start of SoC therapy in MODIFY; however, in this study, patients were on SoC therapy for a median of 11 days before bezlotoxumab infusion. Timely administration of bezlotoxumab infusion can be challenging. Patients were identified as treatment candidates for bezlotoxumab either during an inpatient stay or in the community setting. Hospitalized patients are typically unable to receive therapy until discharge due to formulary restrictions. Community patients must be seen by the physician, provide stool samples, and start SoC therapy. Prior authorization is then required by payors before a bezlotoxumab infusion is scheduled, adding additional days to the time of treatment. Patients in our study who received bezlotoxumab within 7 days after the start of SoC experienced rCDI at a rate comparable to that of those receiving bezlotoxumab >7 days after initiation of SoC (15.2% vs 16.2%). This finding demonstrated that bezlotoxumab efficacy was unaffected by the timing of SoC therapy, as reported previously [22]. The time from bezlotoxumab infusion to rCDI was longer in this study compared with the MODIFY trials, with 52% of CDI recurrences occurring at 4 weeks or later, whereas 71% of the MODIFY population experienced rCDI within 4 weeks. Patient-reported recurrence or delay in diagnosis could have contributed to the difference compared with closely supervised follow-up visits of clinical trial participants. In addition, our study offers no guidance regarding long-term prevention of rCDI beyond 90 days or utility of repeat bezlotoxumab infusions.

Administration of bezlotoxumab was safe, with no serious reactions observed following infusions. This is consistent with MODIFY trials, in which reported adverse drug reactions were similar to placebo [11]. Nevertheless, there is a warning and precaution in the US product insert stating that bezlotoxumab should be reserved for use in patients with underlying CHF when the benefit outweighs the risk [10]. In the MODIFY trials, more patients with a history of CHF died in the bezlotoxumab group vs placebo (19.5% vs 12.5%), with varied causes of death including cardiac failure, infections, and respiratory failure. None of these deaths or reports of CHF were assessed by the investigators to be related to bezlotoxumab or placebo. In this study, 2 of 10 patients with a history of CHF died before the 90-day outcome assessment. Both patients had multiple concurrent comorbidities with adverse events leading to their death 6 and 11 weeks after bezlotoxumab infusion, respectively.

The limitations of this study include the retrospective, nonrandomized study design. The unavailability of *C. difficile* ribotyping limited the ability to correlate recurrence with the CDI strain. Documentation of over-the-counter medications was patient-reported, which may result in an underestimation of gastric acid suppressant use as it was assessed as a potential rCDI risk factor. Additionally, it cannot be ruled out that recurrence rates in patients with rCDI confirmed by PCR were falsely elevated, as suggested in a recent study [25]. It is plausible that some of these patients did not have active disease and were merely colonized with toxigenic strains of *C. difficile* with diarrhea due to other causes; however, all patients in this study were diagnosed and managed by infectious disease physicians or gastroenterologists.

In summary, this real-world multicenter cohort study demonstrated successful prevention of rCDI in 84.1% of patients following administration of a single dose of bezlotoxumab in combination with SoC therapy in US outpatient infusion centers. The CDI recurrence rate was comparable with findings reported in the MODIFY phase 3 trials despite the presence of a more comorbid, at-risk patient population with an extensive CDI history. The type of SoC antibiotic and timing of bezlotoxumab infusion did not affect outcomes in this study. However, multiple prior CDI recurrences were independently associated with a higher risk of subsequent rCDI, supporting earlier use of bezlotoxumab in the disease course.

## Supplementary Data

Supplementary materials are available at *Open Forum Infectious Diseases* online. Consisting of data provided by the authors to benefit the reader, the posted materials are not copyedited and are the sole responsibility of the authors, so questions or comments should be addressed to the corresponding author.

ofaa097_suppl_Supplementary_Tables
