# Supplementary material for: Real-world Experience of Bezlotoxumab for Prevention of Clostridioides difficile Infection: A Retrospective Multicenter Cohort Study
Source: Open Forum Infect Dis. 2020 Mar 19;7(4):ofaa097. doi: 10.1093/ofid/ofaa097 (PMC7186524; doi:10.1093/ofid/ofaa097)
Supplement: ofaa097_suppl_Supplementary_Tables [file ofaa097_suppl_supplementary_tables.docx]

**Supplementary Material**

**Supplementary Table 1. Payor-Specific Coverage Criteria for Administration of Bezlotoxumab (Zinplava™)^a^**

|  | A04.71 - Enterocolitis due to *Clostridium difficile*, recurrent | |
| --- | --- | --- |
|  | A04.72 - Enterocolitis due to *Clostridium difficile*, not specified as recurrent | |
| Patients with ICD-10 codes listed above, who meet ALL criteria below (1 to 5) | | Payor |
| 1 | 18 years of age or older | All |
| 2 | Confirmed diagnosis of CDI with documentation of the following: | All |
|  | Diarrhea (≥3 loose stools in last 24 hours), *and* |  |
|  | Positive stool test for toxigenic *C. difficile* |  |
| 3 | Receiving concomitant SoC antibiotic therapy for CDI | All |
| 4 | At high risk of CDI recurrence, defined as any one of the following: | All |
|  | Age ≥65 years of age or older, *or* |  |
|  | Long-term use of systemic non-CDI antibacterial drugs, *or* |  |
|  | History of ≥1 prior CDI episodes within the previous 6 months, *or* |  |
|  | Immunocompromised, *or* |  |
|  | Clinically severe CDI at presentation (defined as Zar score ≥2), *or* |  |
|  | Hypervirulent strain (ribotypes 027, 078, or 244) |  |
| 5 | No prior treatment with bezlotoxumab for the same active CDI | All |
| Additional payor-specific criteria (6 to 10) | |  |
| 6 | Stool sample must be collected ≤7 days prior to scheduled bezlotoxumab infusion | Aetna, Anthem BCBS (various intermediary) |
| 7 | Stool sample must be collected ≤10 days prior to scheduled bezlotoxumab infusion | BCBS (various intermediary) |
| 8 | Active CDI for ≤14 days prior to receiving scheduled bezlotoxumab infusion and patient has received ≤14 days of SoC antibiotic treatment for CDI | BCBS (various intermediary) |
| 9 | Patient must have confirmed CDI recurrence defined as ≥1 prior CDI episode within the past 6 months | Humana |
| 10 | Patient will receive at least 10 days of antibiotic treatment for CDI and patient has not received a previous dose of bezlotoxumab in the past 6 months | BCBS (various intermediary) |
| Abbreviations: BCBS, Blue Cross Blue Shild; *C. difficile*, *Clostridioides difficile*; SoC, standard of care*.* | | |
| ^a^obtained from medical policy bulletins of respective payor, as of August 05, 2019. | |  |

**Supplementary Table 2. Specific Reasons for Patients not Receiving Bezlotoxumab Following Referral**

| Reason | Results (N=144) |
| --- | --- |
| Payor denial | 65 (45.1) |
| Private insurance | 32 (49.2) |
| Medicare | 28 (43.1) |
| Medicaid | 5 (7.7) |
| Competing problem | 24 (16.7) |
| Treatment of other disease or condition | 9 (6.3) |
| History of congestive heart failure | 7 (4.9) |
| Patient not discharged from hospital | 6 (4.2) |
| Expired prior to scheduled infusion | 2 (1.4) |
| Patient decision | 19 (13.2) |
| Physician decision, other CDI therapy | 15 (10.4) |
| Patient financial hardship | 13 (9.0) |
| Transfer of care | 6 (4.2) |
| Loss to follow-up | 2 (1.4) |
| Data are presented as No. (%). | |
| Abbreviations: CDI, *Clostridioides difficile* infection. |  |
